# Coexpression of *PalbHLH1* and *PalMYB90* Genes From *Populus alba* Enhances Pathogen Resistance in Poplar by Increasing the Flavonoid Content

**DOI:** 10.3389/fpls.2019.01772

**Published:** 2020-02-26

**Authors:** Qiuxian Bai, Bingbing Duan, Jianchao Ma, Yannan Fen, Shujiao Sun, Qiming Long, Jiaojiao Lv, Dongshi Wan

**Affiliations:** State Key Laboratory of Grassland Agro-Ecosystem, School of Life Sciences, Lanzhou University, Lanzhou, China

**Keywords:** *PalbHLH1* and *PalMYB90*, *Populus alba* var. *pyramidalis*, *flavonoid*, *Dothiorella gregaria Sacc*, *Botrytis cinerea*

## Abstract

Secondary metabolites of the flavonoid pathway participate in plant defense, and bHLH and MYB transcription factors regulate the synthesis of these metabolites. Here, we define the regulatory mechanisms in response to pathogens. Two transcription factors from *Populus alba* var. *pyramidalis*, *PalbHLH1* and *PalMYB90*, were overexpressed together in poplar, and transcriptome analysis revealed differences in response to pathogen infection. The transgenic plants showed elevated levels of several key flavonoid pathway components: total phenols, proanthocyanidins (PAs), and anthocyanins and intermediates quercetin and kaempferol. Furthermore, *PalbHLH1* and *PalMYB90* overexpression in poplar enhanced antioxidase activities and H_2_O_2_ release and also increased resistance to *Botrytis cinerea* and *Dothiorella gregaria* infection. Gene expression profile analysis showed most genes involved in the flavonoid biosynthesis pathway or antioxidant response to be upregulated in *MYB90*/*bHLH1*-OE poplar, but significant differential expression occurred in response to pathogen infection. Specifically, expression of *PalF3H* (flavanone 3-hydroxylase), *PalDFR* (dihydroflavonol 4-seductase), *PalANS* (anthocyanin synthase), and *PalANR* (anthocyanin reductase), which function in initial, middle, and final steps of anthocyanin and PA biosynthesis, respectively, was significantly upregulated in *D. gregaria*-infected *MYB90*/*bHLH1*-OE poplar. Our results highlight that PalbHLH1 and PalMYB90 function as transcriptional activators of flavonoid pathway secondary-metabolite synthesis genes, with differential mechanisms in response to bacterial or fungal infection.

## Introduction

The *Populus* species comprises some of the most valuable woody plants in the world due to their rapid growth, easy propagation, adaptation, and wide application in forestation (Polle et al., 2013). However, disease outbreaks in poplar are widespread and threaten poplar plantations and production. Poplar diseases on plantations are mainly induced by fungal or bacterial pathogens that can spread widely in leaves, stems, and even roots, causing widespread poplar death (Ostry and McNabb Jr, 1985; Newcombe and Bradshaw Jr, 1996; Newcombe and Ostry, 2001). Thus, the development of disease-resistant poplar cultivars is becoming necessary and urgent. In general, a successful disease resistance breeding program relies on an understanding of the genetic basis of resistance and interactions between hosts and pathogens, yet genetic information on disease resistance in *Populus* is scarce.

Secondary metabolic products play significant roles in plant resistance to pathogens. Among these metabolites, flavonoids, such as anthocyanins and condensed tannins (also called proanthocyanidins, PAs), accumulate in the seed coat, leaf, stem, bark, and root in response to biotic (Lorenc-Kukuła et al., 2005; Zhang et al., 2013) and abiotic (Dixon et al., 2005; Paolocci et al., 2007) stresses. These molecules also serve as adjuncts in the defense mechanisms of plants (Winkel-Shirley, 2002; Dixon et al., 2005; Koes et al., 2005; Lepiniec et al., 2006) and are involved in the antioxidant response to increase plant resistance to various stresses (Winkel-Shirley, 2002; Dixon et al., 2005). In poplar, PA accumulation can prevent pathogen development by either reducing mycelium growth of infectious fungi (Yuan et al., 2012; Wang et al., 2017) or inhibiting extracellular hydrolases of invading pathogens (Scalbert, 1991). Moreover, a high anthocyanin content can enhance the ROS (reactive oxygen species) scavenging induced by pathogen attack or abiotic stress (Yamasaki et al., 1996). For example, when plants are exposed to UV radiation, flavonols facilitate high antioxidant reactions, and their biosynthesis is upregulated to increase protection against this stress in *Ligustrum vulgare* plants (Agati et al., 2011). Therefore, to uncover the resistance mechanism of flavonoids, their biosynthetic pathways and regulation should be clarified.

The biosynthetic pathways of flavonoids are regulated by many transcription factors, such as R2R3-MYB (Cho et al., 2016; Wang et al., 2017), WD-repeat (WDR), and basic helix-loop-helix (bHLH) transcription factors (Hichri et al., 2011; Huang et al., 2013). In particular, MYB family transcription factors are emerging as central players in the coordinated activation of sets of genes specific to the anthocyanin and tannin pathways (Wang et al., 2017). In poplar, overexpression of MYB19 from *P. trichocarpa* activates expression of *CHS1* (chalcone synthase 1) and *ANS2* (anthocyanins synthase 2), which promotes the accumulation of anthocyanins in hybrid poplar (Cho et al., 2016). In tomato, coexpression of two transcription factors, *Delila* (Del, bHLH) and *Rosea1* (Ros1, MYB), from snapdragon (*Antirrhinum majus*) (Butelli et al., 2008; Maligeppagol et al., 2013) results in accumulation of anthocyanins, which not only extends the shelf life of the transgenic tomatoes but also increase their resistance to *Botrytis cinerea* (Zhang et al., 2013). Anthocyanin production is also commonly induced under stress conditions (Gould, 2004). In tomato, overexpression of *AtMYB12*, *Del*, and *Ros1* increases anthocyanin biosynthetic gene expression and activates primary metabolism (Zhang et al., 2015). In addition, expression of *Del* from snapdragon was shown to enhance leaf and flower anthocyanin production and antioxidant activities in tobacco by regulating the transcript levels of *NtCHS*, *NtCHI*, *NtF3H*, *NtDFR*, and *NtANS* (Naing et al., 2017). Altogether, evidence thus far indicates that overexpression of the Del and Ros1 transcription factors can enhance flavonoid biosynthesis and antioxidant activities and improve resistance to biotic and abiotic stress in transgenic plants. However, it remains unclear whether similar flavonoid biosynthesis response mechanisms to fungal and bacterial infection occurs in poplar.

*Populus alba* var. *pyramidalis* has been widely cultivated in northern China due to its advantageous properties of rapid growth, lack of seed catkins, erect stems, and high biomass production (Xu, 1988; Zhang et al., 2008; Xu et al., 2011). However, wood diseases and insect herbivory occur extensively in *P. alba*, resulting in massive production loss worldwide. Therefore, improving the disease resistance of *P. alba* var. *pyramidalis* using molecular genetic breeding technology is imperative. With the publication of *P. alba* var. *pyramidalis* (Ma et al., 2018) genomic data, screening for candidate genes thought to be involved in the formation of specific poplar traits, such as resistance to pathogens and other stresses, is possible through genome comparison.

In this study, the *PalbHLH1* and *PalMYB90* transcription factors were overexpressed together in *P. alba* var. *pyramidalis*, and accumulation of secondary metabolites from the flavonoid pathway was examined. We also investigated the abilities of the transgenic plants to resist pathogens and analyzed their gene expression profiles. Our findings may be utilized to alter flavonoid biosynthesis *via* metabolic engineering methods to improve poplar resistance to fungal and bacterial pathogens.

## Materials and Methods

### Plant Materials

Two-year-old *Populus euphratica* seedlings and *P. alba* var. *pyramidalis* saplings were collected from Xinjiang Province, China, planted in pots with loam soil, and grown in a semi-greenhouse under a constant temperature of 25°C, a 16/8-h (light/dark) photoperiod and 60% relative air humidity. Saplings and seedlings at similar growth stages were used for pathogen inoculations. *P. alba* var. *Pyramidalis* tissue culture saplings were cultured in WPM, which is a type of woody plant medium (0.1 mg L^−1^ NAA, 400 mg L^−1^ cefotaxime, 9.0 mg L^−1^ hygromycin, 20 g L^−1^ sucrose, and 8.3–8.4 g L^−1^ agar) (Lloyd and McCown, 1980), and plantlets were grown under a 14/10-h (light/dark) cycle with supplemental light (56.25 µmol m^−2^ s^−1^) in a light growth chamber at 25°C.

### *PalbHLH1* and *PalMYB90* Gene Cloning

Total RNA was isolated from the leaves of *P. alba* var. *pyramidalis* saplings using Plant Mini Kit (Qiagen, Germany). First-strand cDNA was synthesized from 2 μg of total RNA in a 20-μl reaction mixture using the RT-AMV transcriptase kit (TaKaRa, Dalian, China). The coding sequences of *PalbHLH1* and *PalMYB90,* were amplified using gene-specific primers (**Table S1** in **Datasheet 1**) designed based on the *PalbHLH1*/*PalMYB90* gene sequences respectively, in the *P. alba* var. *pyramidalis* genome (Ma et al., 2018). PCR was carried out with *Pfu* DNA polymerase (TaKaRa) in a total volume of 50 μl with a thermal cycler program of 98°C for 2 s, 38 cycles of 98°C for 10 s, 55°C for 5 s, and 72°C for 2 min, and a final extension step at 72°C for 10 min. The amplification products were inserted into the plant binary vector pCAMBIA1305 through intermediate vectors pMD19 and pCXSN using the enzyme digestion–linked cloning system to produce *35S::PalbHLH1* and *35S::PalMYB90* constructs (**Figure 2A**). Positive clones were verified by DNA sequencing and aligned with sequences from the *P. alba* var. *pyramidalis* genome (Ma et al., 2018).

### Phylogenetic Analysis

The homologs of *PalMYB90* (*PalRos1*, PAYT030711.1) from other species were retrieved by BLAST searches (http://www.phytozome.com) and aligned using MAGE 5.0 (Tamura et al., 2011) and Genedoc (Lynnon Corporation, USA).The accession numbers are shown in **Table S6** in **Datasheet 1**. The full-length coding sequence of the *PalbHLH1* gene (*PalDel1*, PAYT035597.1) and homologs from other plants (Heim et al., 2003) were downloaded from National Center for Biotechnology Information (https://www.ncbi.nlm.nih.gov) and aligned using MAGE 5.0 (Tamura et al., 2011). The accession numbers are listed in **Table S5** in **Datasheet 1**. Phylogenetic analyses based on amino acid sequences were performed using the neighbor-joining (NJ) and Maximum likelihood (ML) method *via* MAGE 5.0. Phylogenetic analyses were performed using bootstrapping with 1,000 replicates.

### Quantitative Real-Time PCR

Quantitative real-time PCR (qRT-PCR) analysis was performed using a Thermal Cycler Dice Real-Time System TP800 (TaKaRa, Dalian, China) and the specific primers for *PalbHLH1* and *PalMYB90* shown in **Table S3** in **Datasheet 1**. qRT-PCR was performed in a 25 µL reaction volume containing 12.5 µL of SYBR Premix ExTaq™ (Takara, Dalian, China), and the data were analyzed as described by Tsai et al. (2006). *CYC063* was used as the internal reference gene for qRT-PCR (Qu et al., 2016). Differences in gene expression are expressed as the fold change relative to the control, which was calculated using the 2^−△△Ct^ method (Livak and Schmittgen, 2001). Each measurement was carried out in triplicate, and error bars represent the SE of the mean of fold changes for three biological replicates (**Table S1** in **Datasheet 1**). A standard curve was generated using an accurately quantified plasmid containing the target gene and diluted into a series of concentration gradients for the PCR reaction.

### Plant Transformation

*Agrobacterium*-mediated methods (Jia et al., 2010) were used for poplar transformation with some modifications (Ma et al., 2018). One-year-old *P. alba* var. *pyramidalis* clones propagated from cuttings grown in a greenhouse at 25°C under a 16-h light/8-h dark cycle (100 µmol/m^2^/s) were used for transformation. After disinfection with 12% sodium hypochlorite, leaves were cut into pieces and placed on WPM (with 2 mg/L zeatin, 1 mg/L naphthalene acetic acid, 100 µmol/L acetosyringone, 20 g L^−1^sucrose, and 8.3–8.4 g L^−1^ agar) for induction. The poplar leaf discs were infected with recombinant *Agrobacterium tumefaciens* strain GV3101, and putative transgenic plants were selected on WPM supplemented with 9 mg/L hygromycin. The explants were induced to produce new plants under aseptic conditions, which were used for the transformation process performed according to the *P. alba* var. *pyramidalis* transformation protocol (Ma et al., 2018).

### DMACA Staining

To determine PA accumulation, leaves of WT and transgenic lines at the same growth stage were stained for about 20 min with 0.3% (w/v) 4-dimethylaminocinnamaldehyd (DMACA) in a cold mixture of methanol and 6 M HCl (1:1, v/v), rinsed with several changes of 70% (v/v) ethanol and observed under a dissecting microscope. PA-containing cells stained blue, and the stained leaves were preserved in 70% (v/v) ethanol (Li et al., 1996). In brief, after decolorization with 30% acetic acid/methanol for 6–10 h, the leaves were washed two to three times with 75% ethanol and stained with DMACA dye solution for 5–10 min, and the staining results were compared.

### Quantification of Anthocyanin, Quercetin, Kaempferol, Total Phenol, and Tannin Contents

For analysis of anthocyanin levels, 0.5 g of poplar leaves at the same growth stage was ground rapidly in liquid nitrogen; 5 ml of methanol:0.1% ascorbic acid was quickly added to the ground material, and the mixture was sonicated for 1 h. The material was then wrapped in aluminum foil to protect the reaction from light, followed by shaking for 24 h at 120 rpm. After centrifugation at 2,500×*g* for 10 min, 1 ml of the supernatant was mixed with 1 ml ultrapure water, and 1 ml chloroform was added to remove chlorophyll. After centrifugation, the absorbance of the clear supernatant at 530 nm was determined using a spectrophotometer, and the total anthocyanin concentration was calculated using the molar absorbance of cyanidin-3-O-glucoside (Wang et al., 2013): *y* = 0.3305*x* − 0.0024, *R*² = 0.9956 (**Figure S5A**).

Quercetin and kaempferol contents were determined by chromatographic separation using a DIKMA Diamonsil (250 × 4.6 mm, 5 μm) chromatographic column. Fresh leaves (0.1 g) were collected and ground quickly in liquid nitrogen; 1 ml of cold acetone was added, and the mixture was homogenized. After centrifugation at 12,000 rpm for 10 min, the samples were subjected to disintegration *via* ultrasonication for 2 min. The supernatant was mixed with an equal volume of hydrochloric acid and incubated in a water bath at 70°C for 40 min. An equal volume of ethyl acetate was added to the sample, and the mixture was centrifuged at 12,000 rpm for 10 min. The mobile phase was composed of acetonitrile (A) and 0.1% phosphoric acid solution (B) and applied at a flow rate of 1.0 ml/min. Detection was carried out at 360 nm. Finally, the contents of quercetin and kaempferol were determined according to standard curves: *y* = 0.1543*x* + 0.0155 (*R*² = 0.9973) and *y* = 0.1062*x* + 0.006 (*R*² = 0.9981), respectively (**Figures S5B, C**).

The total phenol content was assessed using the folinephenolcolorimetric method (Singleton and Rossi, 1965). Briefly, 100 mg of fresh leaves was ground quickly in liquid nitrogen. After the addition of 1 ml of 80% methanol, the mixture was shaken in the dark for 2 h at 4°C. After centrifugation, the supernatant was diluted with 3 ml of distilled water, after which 0.5 ml of forinol (50% water solution) and 2 ml of a 20% Na_2_CO_3_ solution were added, followed by incubation in a water bath at 45°C for 15 min. After cooling for 10 min, absorbance at 650 nm was measured using a spectrophotometer, and the total phenol content was calculated according to the standard curve of gallic acid: *y* = 0.2791*x* + 0.0086, *R*² = 0.9964 (**Figure S5D**).

For extraction of soluble tannin, 0.5 g fresh leaf tissue was ground in liquid nitrogen and extracted with 5 ml pre-prepared extraction solution (70% acetone and 0.5% ascorbic acid). The samples were ultrasonicated for 1 h and then shaken 24 h in darkness at 20°C. The sample was then centrifuged at 10,000 r/min for 10 min. The supernatant was transferred to another clean centrifuge tube, and excess NaCl was added. After shaking for 5 min, the acetone phase was collected, and the residue was re-extracted twice. The acetone phase was freeze-dried and dissolved in 2 ml methanol containing 0.5% ascorbic acid; 1.5 ml concentrated hydrochloric acid, 3% methanol-soluble vanillin and 0.5 ml final extract were added to a clean test tube for reaction in the dark for 15 min. The absorbance at 550 nm of the final reaction solution was assessed by spectrophotometry to calculate the final content of soluble condensed tannin using a standard curve.

The insoluble tannin content was also measured by spectrophotometry. After extracting soluble tannins, the residue was collected and thoroughly air-dried. Formic acid/butanol (5 ml) was added, and after ultrasonic treatment for 1 h, the absorbance of the reaction solution at 500 nm was measured. After treatment in boiling water for 1 h, absorbance at 500 nm was measured again, and the difference between the two measurements was considered the insoluble tannin content, as calculated using a standard curve. For the tannin standard curve, the preparation method was to absorb 0, 0.1, 0.25, 0.5, 1, and 2 mg/ml tannin standard solution, 1 ml each and placed in the cuvette. Different volumes (0, 0.1, 0.25, 0.5, 1, 2 ml) of the 1 mg/ml standard tannin solution was absorbed to a colorimetric plate. Absorbance was measured at 550 nm, and the standard working curve was drawn. The following regression equation was obtained: *y* = 0.4463*x* + 0.0456, *R* ^2^ = 0.9972 (**Figure S5E**). Each experiment was performed three times with at least three replicate plants per treatment and included controls.

### Examination of Antioxidant Enzyme Activities

To investigate the tolerance of *MYB90*/*bHLH1*-OE poplar plants to various stresses, we evaluated the activities of peroxidase (POD) and superoxide dismutase (SOD) and release of hydrogen peroxide (H_2_O_2_). Wild type (WT), *MYB90*/*bHLH1-2*, and *MYB90*/*bHLH1-5* saplings were grown in soil for 2 months. The eighth leaf from the top was harvested and used to measure SOD and POD activities and the H_2_O_2_ content using a kit (Comin Biotechnology, China) according to the manufacturer’s protocol. Specific operating instructions can be obtained from the company’s website (www.cominbio.com). Each sample included at least three individual plants, and all experiments were conducted in triplicate.

### Transgenic Plant Pathogen Challenge

To evaluate resistance to pathogens, mature leaves of 3-month-old poplar saplings were incubated with *D. gregaria* or *B. cinerea*. *D. gregaria* was grown on potato dextrose agar medium for 3 days at 28°C; *B. cinerea* was grown on malt extract peptone agar medium for 3–5 days at 25°C (Zheng et al., 2006; Huang et al., 2012; Karim et al., 2015). The pathogen-inoculated leaves were covered with a transparent plastic dome to maintain high humidity, and disease development under a constant temperature of 25°C and a 16/8-h (light/dark) photoperiod was evaluated after 5 days. The relative lesion areas of the poplar leaves were evaluated using ImageJ software. Each sample included at least three individual plants, and all experiments were conducted in triplicate.

### Transcriptome Analysis of Transgenic Poplar After Pathogen Infection

One-year-old *P. alba* var. *pyramidalis* saplings were cultivated and infected by the two pathogens under the conditions described above. Three individuals of sapling (WT and *MYB90*/*bHLH1*-OE5) incubated with a pathogen were selected as biological replicates. Total RNA was extracted for transcriptome sequencing using the cetyltrimethyl ammonium bromide (CTAB) procedure (Chang et al., 1993). cDNA libraries were constructed and then sequenced at ANOROAD Genome (Beijing, China) using the Illumina HiSeq 2500 sequencing platform (Illumina Inc., CA, USA) with a 150-bp read length. Image output data from the sequencer were transformed into raw sequence data by base calling; raw data were stored in FASTQ format. Sequence data from this study can be found at Genome Sequence Archive (http://bigd.big.ac.cn/) under accession number CRA001044. Sequenced reads were mapped to the *P. alba* var. *pyramidalis* genome using Tophat2 software, and expression levels were calculated using cufflinks software (Trapnell et al., 2012). Differentially expressed genes (DEGs) were identified by cuffdiff based on the parameters *q* < 0.05 and log_2_ (fold change) ≥1 or ≤ –1. We plotted heatmap based on FPKM through the ggplot2 package (Ginestet, 2011) in R (https://cran.r-project.org/mirrors.html).

### GO and KEGG Enrichment Analyses

Gene Ontology (GO) analysis was classified using Web Gene Ontology Annotation Plot Software (WEGO; http://wego.genomocs.org.cn). The classifications were calculated using custom-designed Perl scripts. The enrichment of the Kyoto Encyclopedia of Genes and genes (KEGG) was carried out using KOBAS 3.0 software (http://kobas.cbi.pku.edu.cn/anno_iden.php), based on homologous genes identified from *P. trichocarpa* using a reciprocity Basic Local Alignment Search Tool (BLAST) analysis (Tuskan et al., 2006; Marchant et al., 2015). In order to prove that the software combination of “Tophat2 and cufflink” is accurate for mapping, gene expression qualification and DEGs screening for the RNA-seq, we repeated the results of D.gregaria group by using Hisat2 for mapping and feature_count for gene expression qualification, and finally Deseq2 and edgeR for DEGs screening. The repeated results were showed in **Supplementary Datasheet 2**.

### Statistical Analysis

Data are presented as the mean ± standard error for each group from three independent experiments. Statistical analysis of the qRT-PCR and secondary metabolite content results was performed using one-way analysis of variance in SPSS 17.0 software (SPSS, Inc., Chicago, IL, USA). The LSD and Duncan tests were applied to compare means and determine significant differences for multiple samples with a significance level *P* = 0.05. We used Origin software (version 8.0) and AI (Adobe Illustrator CC 2017) to plot SPSS statistical data.

## Results

### Isolation and Characterization of the *PalbHLH1* and *PalMYB90* Genes of *P. alba* var. *pyramidalis*

**Figure 1:**
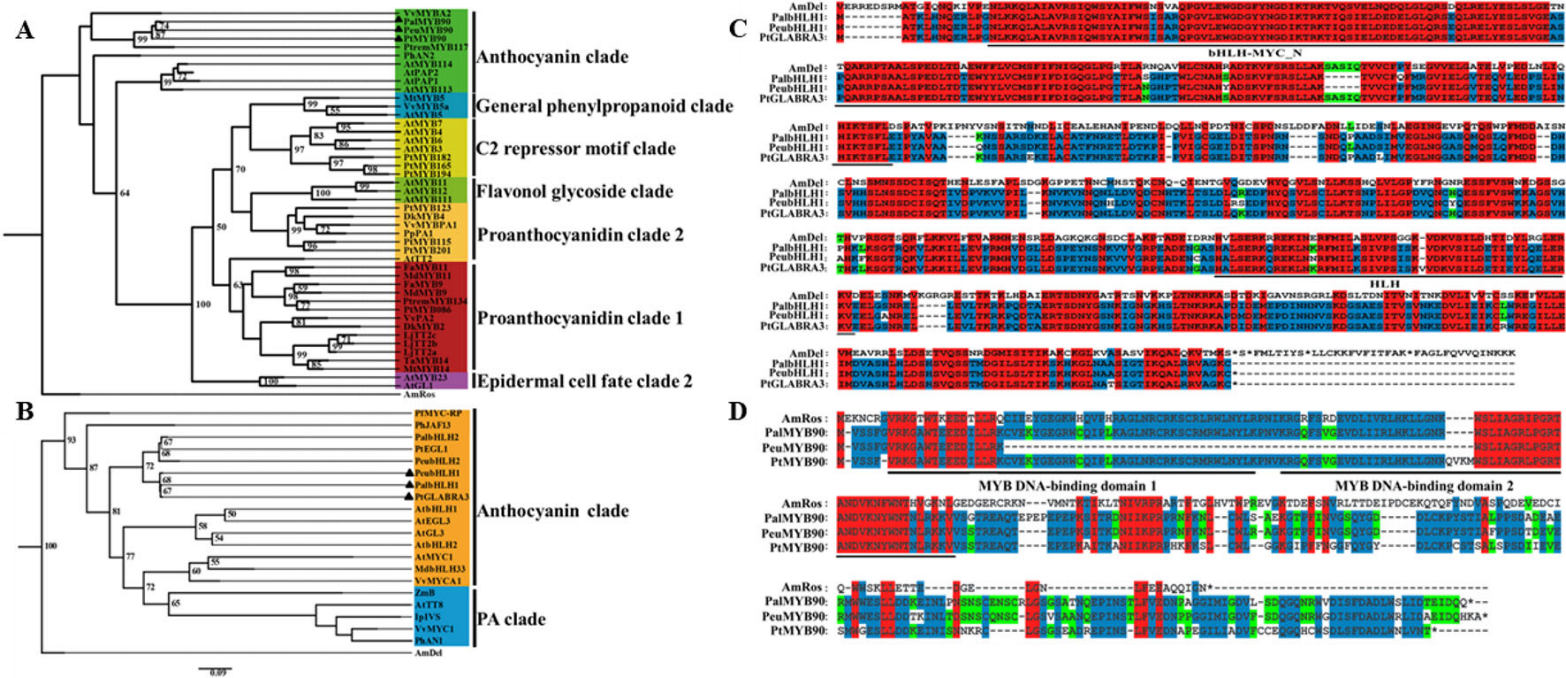
Phylogenetic analyses and multiple alignments of *PalbHLH1* and *PalMYB90*. **(A)** Phylogenetic tree of MYB90 and selected R2R3-Myb proteins from other plant species obtained using the Maximum Likelihood (ML) method in MEGA v.5.1 software. **(B)** Phylogenetic tree of bHLH1 with other bHLH sequences. The scale bar represents 0.05 substitutions per site. **(C**, **D)** The bHLH domains of bHLH1 and R2R3-Myb of MYB90 are both labeled with black lines. Identical amino acids are shaded in red, and similar amino acids are shaded in blue and green.

To investigate the potential mechanisms of the transcription factors PalbHLH1 and PalMYB90 involved in the poplar response to pathogens, we cloned the *PalbHLH1* and *PalMYB90* genes from the *P. alba* var. *pyramidalis* genome (Ma et al., 2018) (**Table S1** in **Datasheet 1**) and aligned them with homologous genes from *P. euphratica* (Ma et al., 2013), *P. trichocarpa* (Tuskan et al., 2006), and *A. majus*. The *PalbHLH1* and *PalMYB90* sequences were identified *via* phylogeny analysis (**Figures 1A, B**) and sequencing with specific primers (**Table S2** in **Datasheet 1**). PalbHLH1 contains a bHLH-MYC-N domain and an HLH domain (**Figure 1C**); PalMYB90 contains two tandem R2R3-Myb DNA-binding domains (**Figure 1D**). All of these protein domains are conserved and similar to their homologs from *A. majus*.

### Construction of Transgenic Poplar Coexpressing *PalbHLH1* and *PalMYB90*

**Figure 2:**
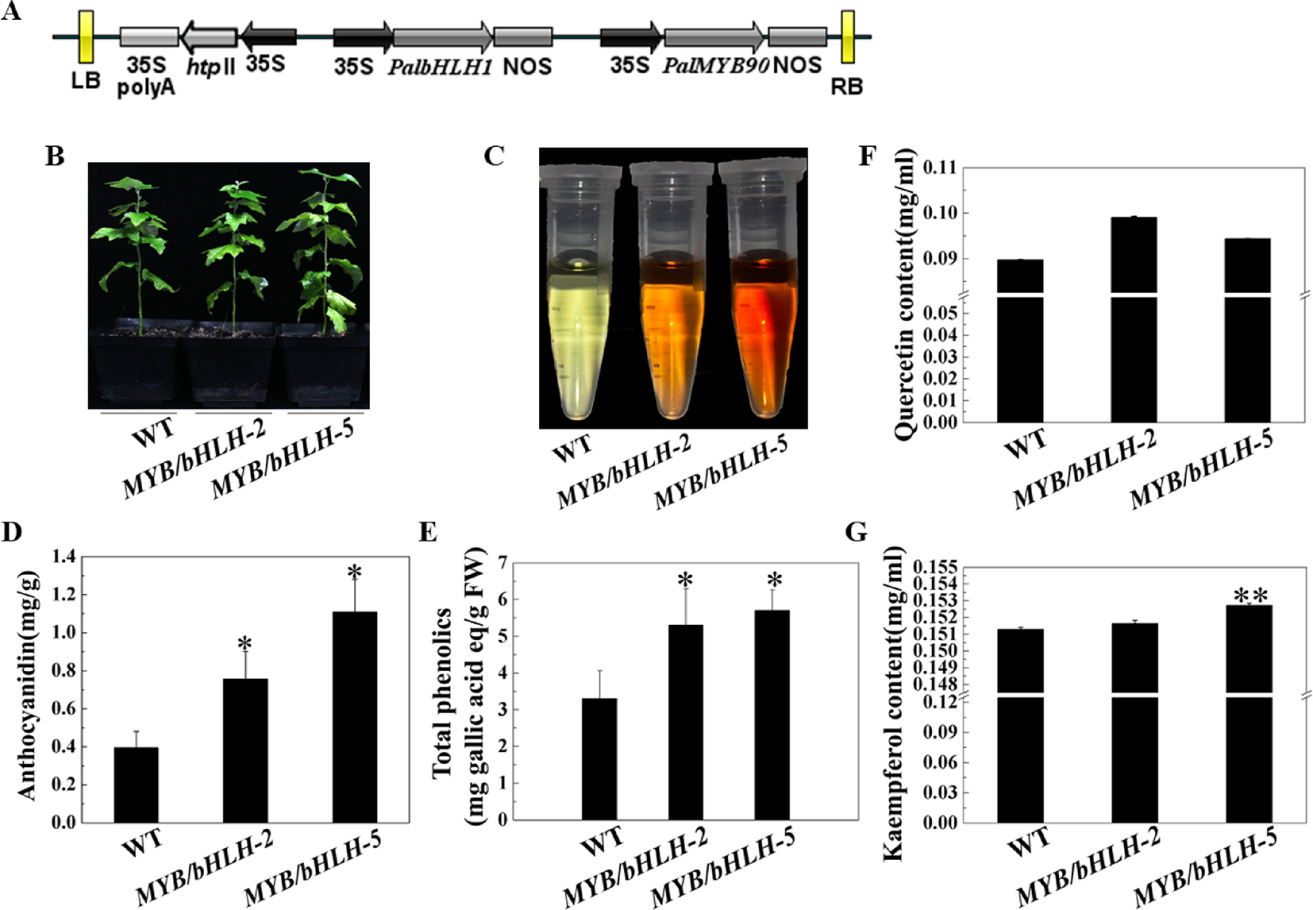
Characterization of transgenic poplar plants. **(A)** Construction of the *MYB90*/*bHLH1*-OE plasmid. **(B)** Growth of transgenic (*MYB90*/*bHLH1*-OE) and wild-type (WT) poplar plants. **(C)** Color of anthocyanin extraction. **(D)** Accumulation of total anthocyanins in the leaves of *MYB90*/*bHLH1*-OE and WT plants. **(E)** Accumulation of total phenolics in the leaves of *MYB90*/*bHLH1*-OE and WT plants. **(F**, **G)** Contents of quercetin and kaempferol in the leaves of *MYB90*/*bHLH1*-OE and WT poplar plants, respectively. The leaves of 2-month-old poplar grown in pots were used in these experiments. Error bars indicate ± SE. Asterisks (*) indicate significant differences, *P* < 0.05. Asterisks (**) indicate significant differences, P ≤ 0.01.

To explore the functions of *PalbHLH1* and *PalMYB90* coexpressed in poplar, the two genes were cloned and ligated into the intermediate vector pCXSN and pMD19, respectively, and the two genes carrying the 35S promoter and NOS terminator were sequentially ligated into the pCAMBIA1305 vector by restriction enzyme ligation, and positive clones were confirmed through sequencing (**Figure 2A**). The expression plasmids were transformed into *P. alba* var. *pyramidalis* leaf discs using the *Agrobacterium*-mediated method as described by Jia et al. (2010), with some modifications (Ma et al., 2018), and two strongly expressing lines, *MYB90*/*bHLH1*-OE2 and *MYB90*/*bHLH1*-OE5, were obtained (**Figure 2B**; **Figures S1**, **S2A**, **S2B** and **Table S3** in **Datasheet 1**). Phenotypic analysis showed no significant differences in the height, weight, or length-to-width ratio of *MYB90*/*bHLH1*-OE2 or *MYB90*/*bHLH1*-OE5 leaves compared to those of WT poplar (**Figures S2C–E**). However, compared with WT leaves, the anthocyanin content of *MYB90*/*bHLH1*-OE was 1.9- to 2.8-fold higher (*P* < 0.05, **Figures 2C, D**) and the quercetin and kaempferol contents of *MYB90*/*bHLH1*-OE were1- to 1.1-fold higher (*P* < 0.01, **Figures 2F, G**). The total amount of flavonoids containing phenol rings (i.e. the total phenol content) in *MYB90*/*bHLH1*-OE2 and *MYB90*/*bHLH1*OE5 leaves was 1.6- to 2-fold higher than that in WT leaves (*P* < 0.05, **Figure 2E**). Interestingly, *PalbHLH1* expression in the *MYB90*/*bHLH1*-OE5 line was up to 1400-fold higher than that in the WT plant (**Figure S2B**), and its expression in the *MYB90*/*bHLH1*-OE2 line was 130 times higher than that in the WT plant (**Figure S2B**). This increase affected the accumulation of anthocyanids, tannins, and other secondary metabolic products in the leaves of the two overexpressing lines. Moreover, the flavonoid content in *MYB90*/*bHLH1*-OE5 was 0.2- to 0.5-fold higher than that in *MYB90*/*bHLH1*-OE2. These results indicate that overexpression of *PalbHLH1* and *PalMYB90* enhances the accumulation of secondary metabolites in the flavonoid pathway.

### Coexpression of *PalbHLH1* and *PalMYB90* Genes Increases the Antioxidant Capacities of Transgenic Poplar

**Figure 3:**
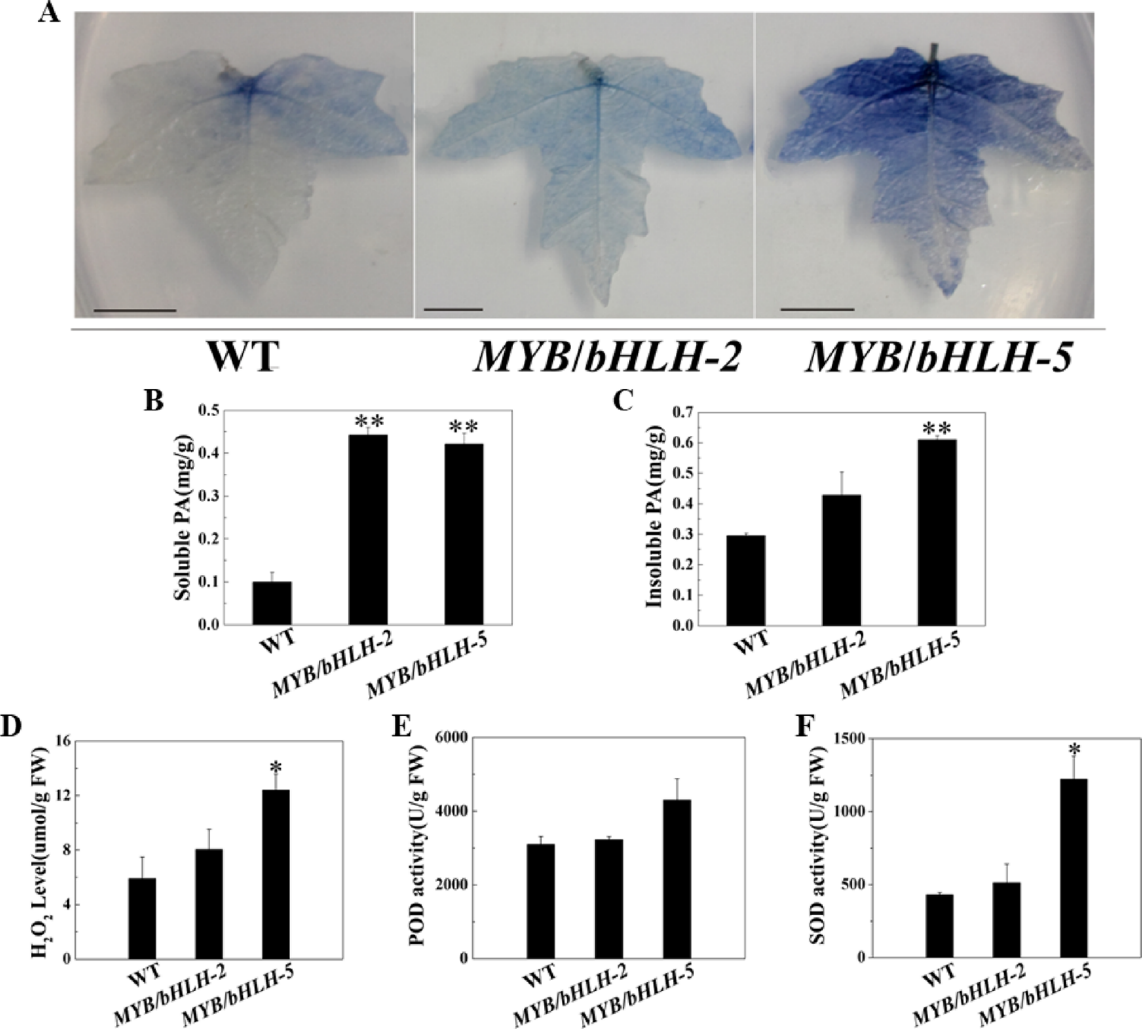
Quantification of insoluble and soluble tannin (PA) and reactive oxygen species in wild-type (WT) and transgenic lines (*MYB90*/*bHLH1*-OE 2 and *MYB90*/*bHLH1*-OE 5). **(A)** PA accumulation detected by DMACA staining. **(B)** Quantification of soluble PA. **(C)** Quantification of insoluble PA. **(D)** Changes in H_2_O_2_ content and **(E, F)** activities of two antioxidases, POD and SOD, in *MYB90*/*bHLH1*-OE poplar leaves. Values represent the means of three replications. Error bars indicate ± SE. Asterisks indicate significant differences: *P < 0.05. Asterisks (**) indicate significant differences, P ≤ 0.01.

To study antioxidant capacities, we first measured the content of tannins, which has been confirmed to enhance the antioxidant capacity of plants (Li et al., 2007). Leaves of transgenic and WT plants were stained with DMACA, which reacts specifically with tannins and flavan-3-ols to form a blue chromophore (**Figure 3A**). As expected, the intensity of DMACA staining of WT leaves was reduced compared to that of the overexpressing lines, indicating a higher concentration of PAs in the leaves of the latter. These results were confirmed by comparing tannin levels in leaves of control and OE lines, and compared to WT, two transgenic lines (OE2 and OE5) showed significantly increased contents (*P* < 0.05) of both soluble and insoluble PA in their leaves (**Figures 3B, C**).

To further prove that the transgenic plants have stronger antioxidant capacity, we determined POD/SOD activities and the H_2_O_2_ content. H_2_O_2_ levels were increased according to *PalbHLH1* and *PalMYB90* expression levels (**Figures 3D–F**). In addition, the activity of SOD was markedly increased in *MYB90*/*bHLH1*-OE5 compared to that in WT and *MYB90*/*bHLH1*-OE2 (*P* < 0.05). Interestingly, POD activity did not differ among WT, *MYB90*/*bHLH1*-OE2, and *MYB90*/*bHLH1*-OE5 plants (*P* > 0.05). A high anthocyanin content can enhance the ROS scavenging induced by pathogen attack or abiotic stress (Yamasaki et al., 1996), and the two transgenic lines (*MYB90*/*bHLH1*-OE2 and *MYB90*/*bHLH1*-OE5) exhibited significantly increased anthocyanin contents, as described above. Therefore, we suspect that the content of ROS was not only affected by the content of anthocyanids in the overexpressing plants; that is, the accumulation of H_2_O_2_ was not regulated solely by anthocyanins. Overexpression of *PalbHLH1* and *PalMYB90* itself may also affect ROS accumulation in *MYB90*/*bHLH1*-OE lines to aid in plant disease defense (Cui et al., 2018). In fact, accumulation of H_2_O_2_ and other ROS is one of the earliest events in host-pathogen recognition, and it has been postulated to play an important role in plant defense (Baker and Orlandi, 1995). Accordingly, elevated SOD and POD help to coordinate H_2_O_2_ accumulation to control the effects of excessive ROS on plants. Together, the elevated levels of antioxidants in the transgenic plants reduced the tissue-damaging effect of ROS and thus likely increased resistance in the *MYB90*/*bHLH1*-OE2 and *MYB90*/*bHLH1*-OE5 lines. These results indicate that overexpression of *PalbHLH1* and *PalMYB90* can enhance the PA concentrations and antioxidant capacity in transgenic poplar.

### *PalbHLH1/* *PalMYB90* Overexpression Enhances Resistance to *D. gregaria* and *B. cinerea* in Transgenic Poplar

**Figure 4:**
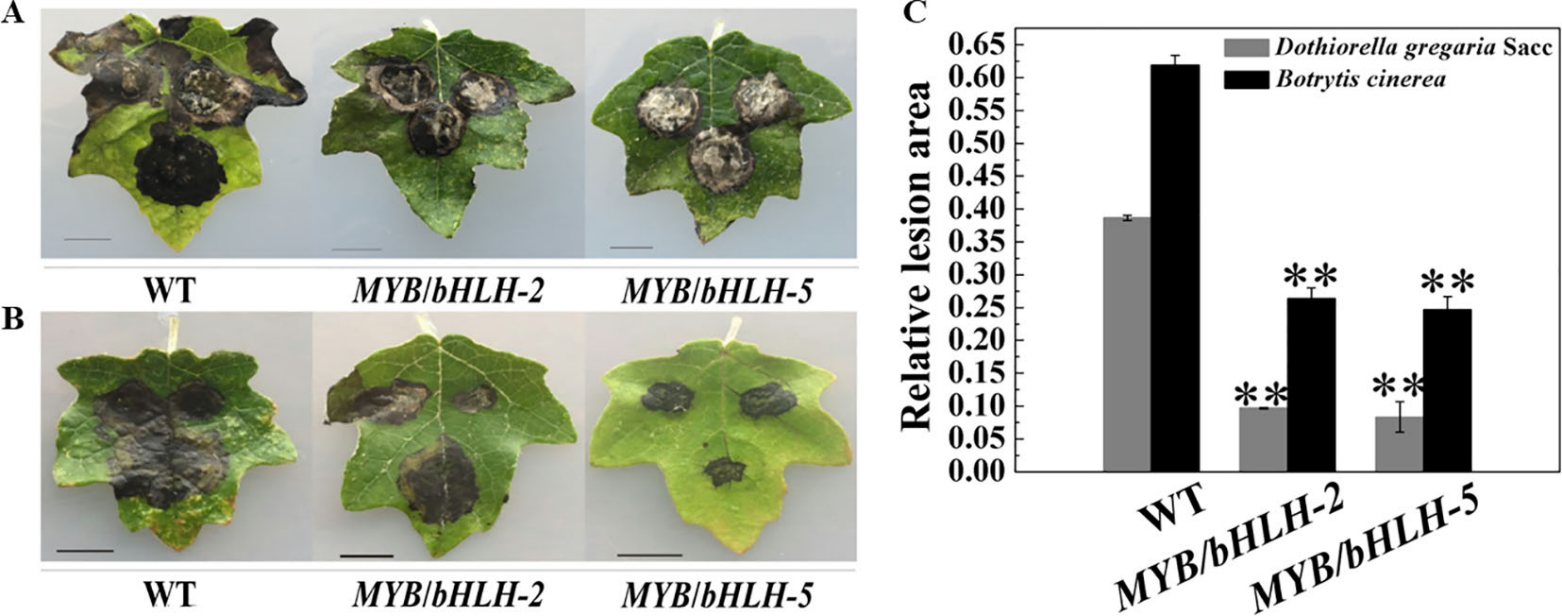
Resistance of transgenic poplar to pathogen infection. Disease symptoms of three leaves of wild-type (WT) and *MYB90*/*bHLH1*-OE plants after *B. cinerea* infection for 4 days **(A)** or *D. gregaria* infection for 7 days **(B)**. **(C)** Ratios of the lesion areas of infected leaves from WT and *MYB90*/*bHLH1*-OE plants (OE-2 and OE-5 lines). Values represent the means of three replications. Error bars indicate ± SE. Asterisks indicate significant differences: **P ≤ 0.01.

Poplar plants serve as hosts for fungal pathogens, such as *Aspergillus niger* and *D. gregaria* (Scalbert, 1991; Yuan et al., 2012), and bacterial pathogens. To determine the initial effects of *PalbHLH1*/*PalMYB90* overexpression with regard to pathogen resistance, we compared the relative lesion areas of infected leaves. The leaves of transgenic and WT plants were inoculated with *D. gregaria* and *B. cinerea* hyphal suspensions for 5–8 days. Regardless of whether *D. gregaria* or *B. cinerea* was applied, the lesion area of the WT lines was two times larger than those of the overexpression lines (**Figures 4A, B**), indicating that *MYB90*/*bHLH1*-OE2 and *MYB90*/*bHLH1*-OE5 had significantly stronger ability to resist both pathogens than did WT (*P* < 0.01). To further visualize the degree of leaf infection by pathogens, we quantitatively analyzed the size of the lesion areas using ImageJ software, with the *MYB90*/*bHLH1*-OE lines only 25–30% of the WT- *D. gregaria* lesion areas and 35–45% of the WT- *B. cinerea* lesion areas (**Figure 4C**). However, no significant differences between *MYB90*/*bHLH1*-OE2 and *MYB90*/*bHLH1*-OE5 lesion areas were observed (**Figures 4A–C**). We speculate that although the expression levels of *PalbHLH1* were significantly different between the two transgenic lines and the flavonoid contents also differed, their abilities to resist disease did not differ after a certain point.

### Determination of Gene Expression Induced by *PalbHLH1*/*PalMYB90* and Pathogen Infection

To investigate genes related to the improved pathogen resistance of the transgenic poplar, we analyzed gene expression profiles in transgenic lines and after infection by two pathogens. The results showed most of the genes involved in the biosynthesis of anthocyanins and flavonoids were upregulated in the transgenic plants, especially after infection. The results showed most of the genes involved in the biosynthesis of anthocyanins and flavonoids were upregulated in the transgenic plants, especially after infection (**Supplementary Datasheet 3**). In brief, *PalbHLH1* and *PalMYB90* expression levels were upregulated in the transgenic lines, and both genes were expressed at particularly higher levels after infection by the two pathogens (**Figures S3A, B**). Transcriptome analysis showed that overexpression of *PalbHLH1*/*PalMYB90* promoted expression of genes involved in flavonoid biosynthesis, such as those encoding fasciclin-like arabinogalactan 6 (FLA), MYB 46, POD, laccase/diphenol oxidase family protein, WRKY 70, NAC 12, ERF 1 (ethylene responsive factor), and basic chitinase (**Figures S3C, D**).

**Figure 5:**
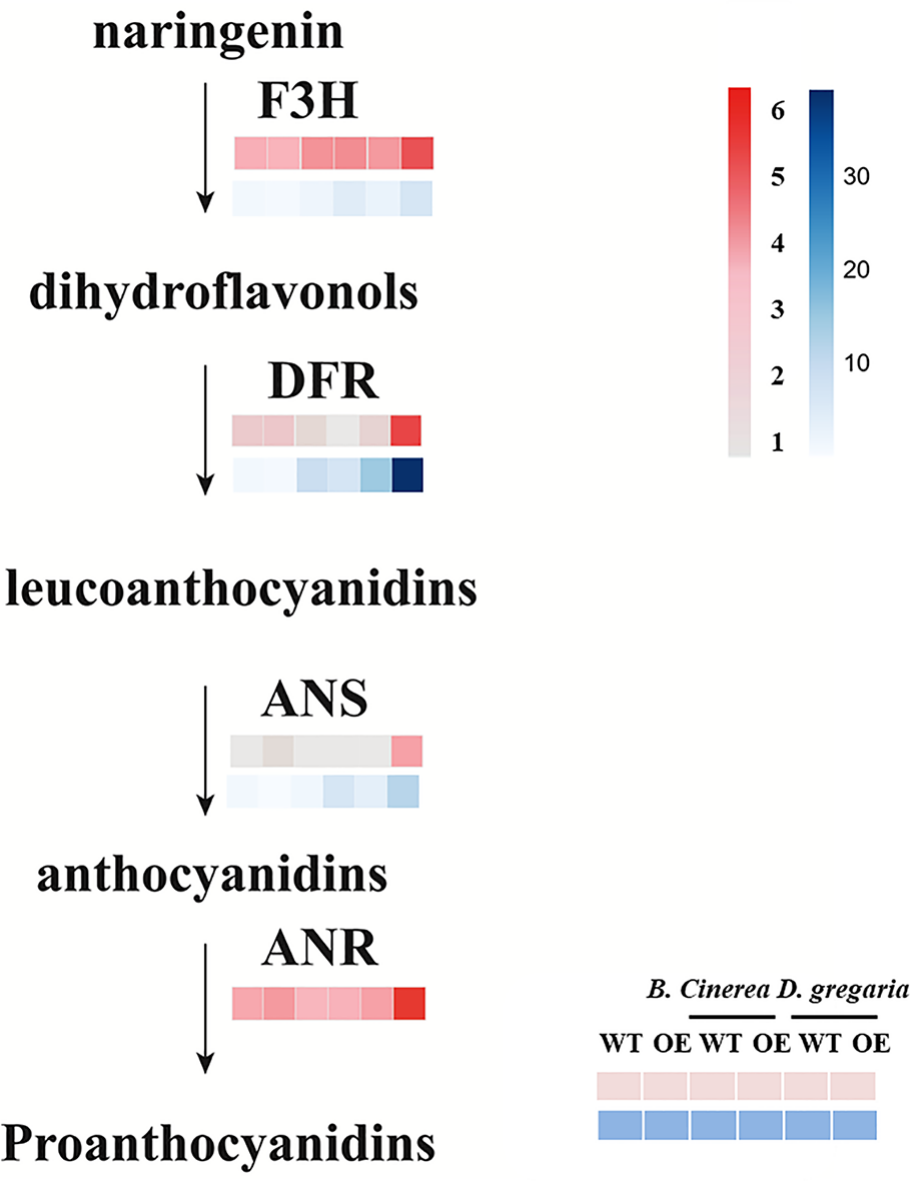
Expression patterns of flavonoid pathway genes in transgenic and control plants infected by two pathogens. Among them, pink blocks are gene expression patterns based on transcriptome data analysis, while blue blocks are qRT-PCR validation results for these genes. Error bars indicate ± SE.

In total, 56 genes, including those encoding the transcription factors MYB113, ERF4 and ERF5, and bHLH factors, were differentially expressed after infection by *D. gregaria* or *B. cinerea* (**Figures S3E, F**), though responses to *D. gregaria* and *B. cinerea* differed. After infection by *D. gregaria*, genes coding for several key enzymes involved in the regulation of secondary metabolite processes and biosynthesis, such as CHS (chalcone synthase), chalcone and stilbene synthase (PAYT009441.1), F3H (flavanone 3-hydroxylase), and DFR (dihydroflavonol 4-reductase), were significantly upregulated in *MYB90*/*bHLH1*-OE lines compared with in control plants (**Figures S4A–C**). Conversely, no differences among these genes were found after infection by *B. cinerea*. Pathogen infection might have induced differential expression of only 17 genes, including *PalbHLH1*, two late embryogenesis abundant (*LEA*) genes and the gene encoding glucosyl transferase 8 (**Figures S4D–F**). Furthermore, with reference to the latest research (Yu et al., 2019), we focus on the expression levels of genes coding for PalF3’H, PalDFR, PalANS, and PalANR, which catalyze the initial, middle, and final steps of anthocyanin and PA biosynthesis, respectively, were significantly upregulated in *MYB90*/*bHLH1*-OE poplar after infection by *D. gregaria* but not after infection by *B. cinerea* (**Figure 5**).

**Figure 6:**
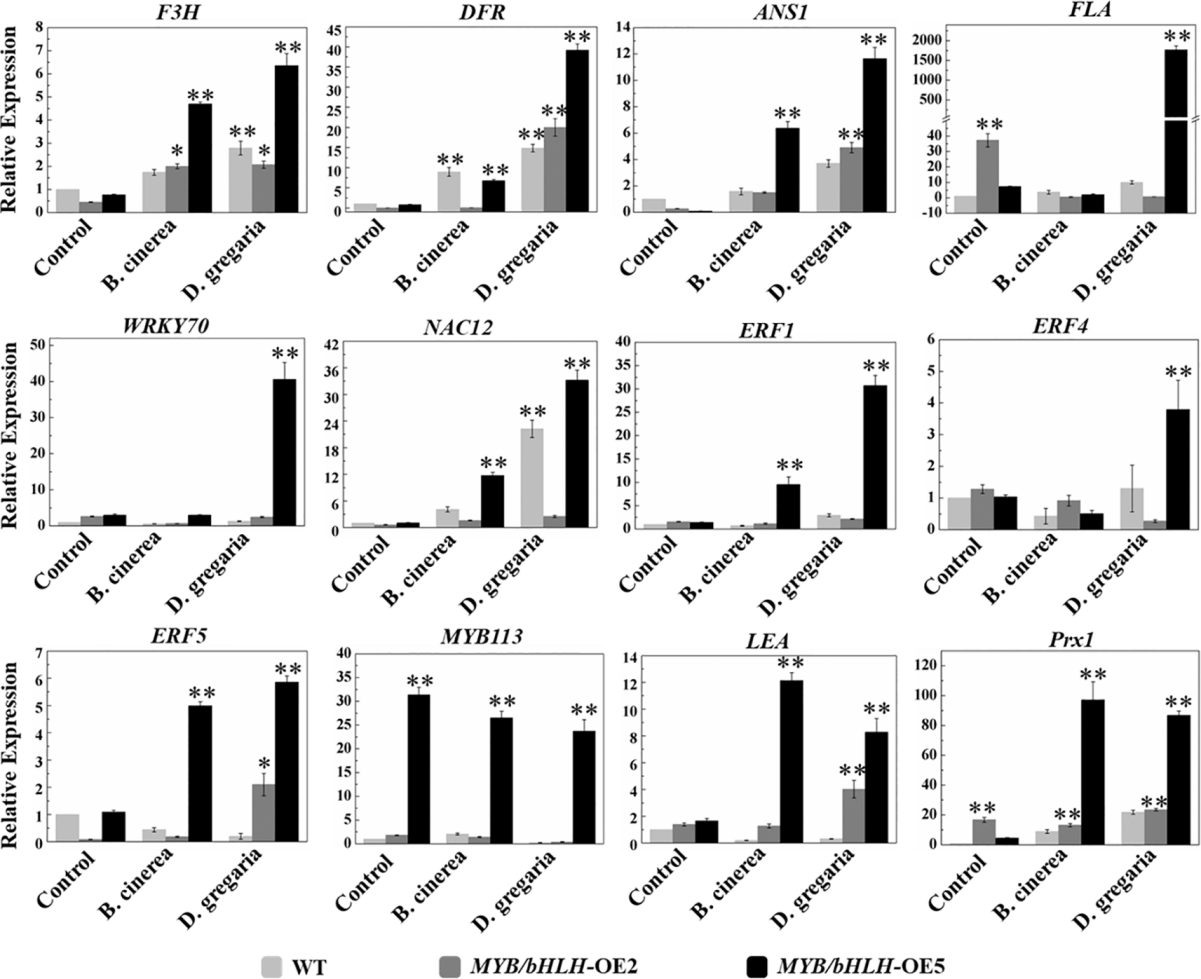
Quantitative real-time PCR analysis of the transcript levels in WT and both independent *MYB90*/*bHLH1*-OE lines before and after being infected by two types of pathogens. The *CYC063* expression was set as the internal control. In the figure, light gray bar represents WT, dark gray and black bars represent *MYB90*/*bHLH1*-OE2 and *MYB90*/*bHLH1*-OE5 lines, respectively. Each measurement was carried out in triplicate, and error bars represent the SE of the mean of fold changes for three biological replicates. Asterisks indicate significant differences: *P < 0.05, **P ≤.01.

To further validate the transcriptome results, we selected 12 genes related to flavonoid synthesis for qRT-PCR analyses in both MYB90/bHLH1-OE poplar lines (OE-2 and OE-5): *F3H, DFR, ANS1, FLA, WRKY70, NAC12, ERF1, ERF4, ERF5, MYB113, LEA*, and *Prx1* (encoding class III peroxidase). The results showed that the expression levels of the *PalF3’H*, *PalDFR*, and *PalANS* genes in two transgenic lines were generally higher than those in wild type after infection by the two pathogens. Most of the 12 genes of the OE-5 line examined here, showed basically similar expression patterns with that of in RNA-seqs (**Figure 6**, **Table S4** in **Datasheet 1**).

### Anthocyanins Enhance the Resistance of *P. euphratica* to *D. gregaria* Infection

**Figure 7:**
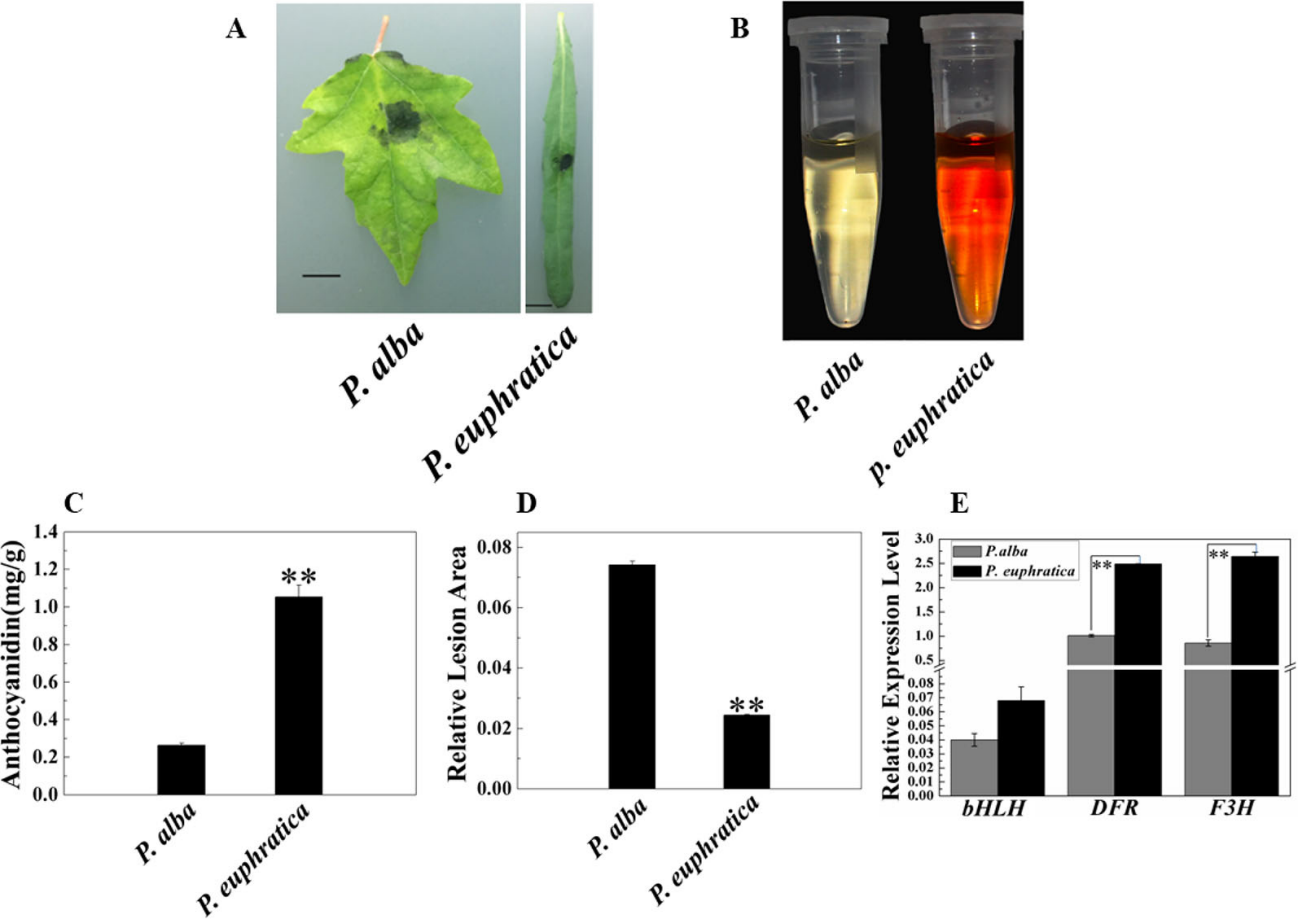
Relationships of anthocyanin content and responses to pathogen infection in *P. alba* var. *pyramidalis* and *P. euphratica* leaves. **(A)** Disease symptoms induced by *D. gregaria* on the leaves of two sister poplars, *P. alba* var. *pyramidalis* and *P. euphratica*. **(B)** Colors of anthocyanin extracted from *P. alba* var. *pyramidalis* and *P. euphratica* leaves. **(C)** Total anthocyanin contents in *P. alba* var. *pyramidalis* and *P. euphratica* leaves. **(D)** Relative lesion areas of the third leaves of *P. alba* var. *pyramidalis* and *P. euphratica* plants after *D. gregaria* infection for 7 days. **(E)** Relative expression levels of *bHLH1*, *DFR*, and *F3H* in *P. alba* var. *pyramidalis* and *P. euphratica* leaves. Error bars indicate the standard error (SE) of the average anthocyanin content, **P < 0.01.

In addition to studying the mechanism of enhanced resistance to fungal pathogens in *P. alba* var. *pyramidalis*, we were also interested in the disease resistance and mechanism of different poplar varieties. Therefore, we compared the pathogen resistance abilities of *P. euphratica* and *P. alba* var. *pyramidalis* by infecting the leaves of saplings of both with *D. gregaria*. Both poplar species are closely related species but show significant difference either in resistance to pathogens and abiotic stresses or morphology (Ma et al., 2013; Ma et al., 2018). The relative lesion area of *P. euphratica* leaves was significantly smaller than that of *P. alba* var. *pyramidalis* leaves (*P* < 0.05) (**Figures 7A, D**), indicating that the former had a greater ability to resist *D. gregaria* infection. The anthocyanin content in *P. euphratica* leaves was also significantly higher than that in *P. alba* var. *pyramidalis* leaves (*P* < 0.05) (**Figures 7B, C**), and the expression level of *PalbHLH1* in *P. euphratica* (*PeuGLABRA*3-1) was higher than that in *P. alba* var. *pyramidalis* (*P* < 0.05) (**Figure 7E**). We also tested the expression level of the structural genes of anthocyanin pathway for *P. euphratica* and *P. alba* var. *pyramidalis*, such as *F3H* and *DFR*. The results showed that the expression level of these genes in *P. euphratica* was higher than that in *P. alba* var. *pyramidalis* (**Figure 7E** and **Table S7** in **Datasheet 1**). However, the expression levels of *PalMYB90* in the two species were lower than the limit of detection. In general, compared to *P. alba* var. *pyramidalis*, *P. euphratica* displayed higher resistance to *D. gregaria.*

## Discussion

### *PalbHLH1* and *PalMYB90* Overexpression Enhances Accumulation of Secondary Metabolites in the Flavonoid Pathway

Secondary metabolites in the flavonoid pathway are involved in responses to various stresses (Lepiniec et al., 2006), and their production is regulated by a series of transcription factors, including MYB, basic bHLH, and WD-repeat proteins (Baudry et al., 2004; Chezem and Clay, 2015), acting on related genes. Transgenic expression of anthocyanin-regulating transcription factors in ornamental plants leads to accumulation of anthocyanin products in other plants (Nishihara and Nakatsuka, 2011). For example, anthocyanin production was enhanced in tomato by coexpression of the transcription factors Del and Ros1 derived from snapdragon (*A. majus*) (Butelli et al., 2008; Maligeppagol et al., 2013). *Del* and *Ros1* overexpression also increases expression of genes related to anthocyanin biosynthesis (Zhang et al., 2015; Ullah et al., 2017; Wang et al., 2017). These findings provide a plausible strategy and model for improving the disease resistance of plants to different pathogenic microorganisms. In this study, we would like to investigate whether over-expression of orthologous can improve disease resistance in poplar, and whether the resistant mechanisms are consistent with in that of in tomato. We overexpressed the two endogenous poplar genes involved in flavonoid metabolism and assessed disease resistance of the poplar species with the weakest resistance. Coexpression of *PalbHLH1* and *PalMYB90* from *P. alba* var. *pyramidalis* was driven by two 35S promoters in transgenic *P. alba* var. *pyramidalis*. Overexpression of *PalbHLH1* and *PalMYB90* not only promoted accumulation of secondary metabolites, such as anthocyanins and PAs, but also enhanced the contents of their intermediate products quercetin and kaempferol. PAs are effective molecules in chemical defense against foliar rust infection in poplar (Ullah et al., 2017). Depending on the level of anthocyanin accumulation, the total polyphenol, soluble PA, and insoluble PA contents increased with *PalbHLH1* and *PalMYB90* expression levels across transgenic poplar lines. From this perspective, the regulation of anthocyanin accumulation affects not only the colors of plant flowers and fruits but also the biosynthesis of secondary metabolic products in other tissues, such as leaves, roots, and stems, all contributing to improving plant resistance to pathogens (Zhang et al., 2013). This study has important implications for environmental restoration.

### *PalbHLH1* and *PalMYB90* Overexpression Induces Activation of Regulatory Pathways Involved in Pathogen Resistance

Secondary metabolite accumulation in the flavonoid pathway contributes to improving plant pathogen resistance. For example, PA accumulation can reduce the growth of mycelia of fungi infecting poplar (Yuan et al., 2012; Ullah et al., 2017; Wang et al., 2017) or inhibit extracellular hydrolases of invading pathogens (Scalbert, 1991); when accumulated at high levels, anthocyanins act as ROS scavengers to reduce the oxidative bursts induced by pathogen infection (Yamasaki et al., 1996). Together, these factors decrease the damage caused by pathogen infection in plants. The synthesis of these metabolites is regulated by a series of complex pathways that are involved in plant growth, development, and responses to environmental stimuli (Butelli et al., 2008; Maligeppagol et al., 2013). Compared with tomato, Del and Ros (*PalbHLH1* and *PalMYB90*) of poplar exhibit functional novelty. Transcriptome analysis showed that overexpression of *PalbHLH1* and *PalMYB90* promoted expression of many transcription factors associated with biosynthesis in the flavonoid pathway, which is involved in pathogen resistance. For example, *MYB113*, a homologous gene of *MYB115*, which encodes a member of the MYB transcription factor family, affects the expression of enzymes involved in later steps of anthocyanin biosynthesis, such as CHI1, CHS4, and DFR1, and leads to anthocyanin accumulation in potato (Liu et al., 2016) and PA accumulation in poplar (Wang et al., 2017). These findings suggest that MYB113 directly or indirectly regulates these genes.

Furthermore, pathogen infection induced expression of key genes in the flavonoid pathway. For example, after *D. gregaria* infection, Gene Ontology (GO) annotations of DEGs included mainly oxidoreductase activity, secondary metabolite processes, and intracellular parts. DFR is an important enzyme that catalyzes the conversion of dihydroflavonol into leucoanthocyanidin, which can be further transformed into anthocyanin and PA, respectively (Dixon et al., 2013). F3H and FLS, two important enzymes controlling the biosynthesis of the key intermediate products quercetin and kaempferol, were also induced by pathogen infection (Lepiniec et al., 2006), which led to more quercetin and kaempferol accumulation in transgenic lines than in WT plants. The genes encoding other enzymes involved in the anthocyanin and flavonoid pathways, such as leucoanthocyanidin dioxygenase, chalcone and stilbene synthase (PAYT005204.1), WRKY75, WD40 domain protein, glutathione S-transferase 19 (GST19), and flavin-dependent monooxygenase 1, were also upregulated. Among them, the WD40 protein activates early biosynthetic genes of the flavonoid pathway (e.g. *F3H*, *DFR*, *ANS*, *ANR*) as well as genes that are specific to the anthocyanins pathway (e.g. *ANS* and *UFGT*) (reviewed by Dixon et al., 2013). The expression levels of *PalF3H*, *PalDFR*, *PalANS*, *PalANR*, which function in the initial, middle, and final steps of anthocyanin and PA biosynthesis, respectively, were significantly upregulated in *MYB90*/*bHLH1*-OE poplar after *D. gregaria* infection.

After *B. cinerea* infection, only 17 genes were differentially expressed. One was the gene encoding glucosyl transferase 8, which is responsible for the biosynthesis of flavone O-glucosides in the flavonoid pathway and improves tolerance to UV radiation and drought (Peng et al., 2017). Compared with the DEGs from *MYB90*/*bHLH1*-OE poplar infected by *D. gregaria*, the related genes after infection by *B. cinerea* expressed no significantly difference in transcriptome analysis. Thus, we performed qRT-PCR to verify expression levels of the DEGs regulated by two pathogen infection (**Figure 6**). The results showed that expression of most of the DEGs were different significantly and positively correlated with the results of the phenotype (**Figure 4**). These results suggest that the *MYB90*/*bHLH1*-OE lines responded differently to *B. cinerea* and *D. gregaria* infection. Moreover, expression of genes encoding some antioxidants or antioxidases, such as SOD, POD, and laccase/diphenol oxidase, was enhanced by *PalbHLH1*/*PalMYB90* overexpression. These results indicate that the accumulation of secondary metabolites, especially anthocyanins, is likely associated with higher antioxidant activities (Fini et al., 2011; Qiu et al., 2014). Interestingly, the content of H_2_O_2_ also increased significantly in the transgenic lines; however, because the content of H_2_O_2_ itself was low, the final H_2_O_2_ content was low even though the content increased significantly. Therefore, we speculate that the content of H_2_O_2_ is not only affected by flavonoids and the activities of antioxidant enzymes but may also be related to overexpression of *PalMYB90* and *PalbHLH1*. Indeed, overexpression of *MYB90* and *bHLH1* genes is a type of stress itself when compared with WT. It has been shown that lower levels of H_2_O_2_ play an important role in plant defense, and we suggest that lower levels of H_2_O_2_ in transgenic plants also contribute to plant defense against pathogens.

### *PalbHLH1* Expression Improves Poplar Resistance to Pathogen Infection

Metabolic responses to specific stressors are conserved among flowering plants. For example, the pathways of antimicrobial compound biosynthesis in response to pathogen infection are conserved (Mansfield, 2000; Dixon, 2001). Thus, comparing stress responses among closely related species may help in the elucidation of candidate genes involved in specific stress responses and the formation of adaptive traits. In this study, we compared the expression levels of *bHLH1*, *F3H*, and *DFR* and the anthocyanin content in leaves infected with *D. gregaria* between *P. euphratica* and *P. alba* var. *pyramidalis*. We found that both *bHLH1*, *F3H*, and *DFR* expression and anthocyanin accumulation were induced more rapidly in *P. euphratica* than in *P. alba* var. *pyramidalis*. In addition, the relative lesion area in *P. euphratica* leaves was smaller than that in *P. alba* var. *pyramidalis* leaves, indicating that the higher anthocyanin accumulation in *P. euphratica* leaves, positively regulated by *bHLH1* might contribute to improve its resistance to *D. gregaria*. *P. euphratica* prefers to grow in arid or semiarid environments and has high resistance to drought and salinity (Zhang et al., 2008; Ma et al., 2013). Therefore, high *PalbHLH1* expression levels in combination with anthocyanin accumulation in *P. euphratica* should enhance its tolerance to severe conditions (Fini et al., 2011; Nakabayashi et al., 2014; Naing et al., 2017).

### Application of *PalbHLH1*/*PalMYB90* Overexpression to Improve Pathogen Resistance in Poplar

*P. alba* var. *pyramidalis* has been widely cultivated for urban afforestation and ecological restoration from northwest to northern China because of its advantageous traits of rapid growth, lack of seed catkins, erect stems, and high biomass production (Xu, 1988; Zhang et al., 2008; Xu et al., 2011; Ma et al., 2018). Nonetheless, poplar plants, particularly *P. alba* var. *pyramidalis*, are easily infected by numerous pathogens, such as *B. cinerea*, *D. gregaria*, and *Melampsora larici-populina* (Miranda et al., 2007; Yuan et al., 2012). Thus, breeding poplars for disease resistance is becoming increasingly urgent and necessary. In this study, we found that overexpression of *PalbHLH1* and *PalMYB90* enhanced the accumulation of secondary metabolites, resulting in improved resistance to bacterial/fungal pathogen infection. Our study provides an efficient genetic engineering strategy for increasing the accumulation of secondary metabolic products in transgenic poplar plants to enhance their capacity of pathogen resistance.

## Conclusion

In summary, Overexpression of *PalbHLH1*/*PalMYB90* led to enhanced secondary metabolic accumulation and antioxidase activities, which all contribute to enhance the resistance of poplars to pathogen infection.

## Data Availability Statement

The datasets generated for this study can be found in the Genome Sequence Archive website (http://bigd.big.ac.cn/) under accession number CRA001044.

## Author Contributions

DW supervised the project. QB and BD performed the experiments. QB, JM and YF participated in analyzing the transcript data. SS, YF, QL and JL provided assistance in the experiments and transcript analysis. All authors read and approved the final manuscript.

## Funding

The research was supported by the National Science Foundation of China (No. 31870580) and the Fundamental Research Funds for the Central Universities (lzujbky-2017-k14).

## Conflict of Interest

The authors declare that the research was conducted in the absence of any commercial or financial relationships that could be construed as a potential conflict of interest.

## Supplementary Material

Figure S1 Validation of agarose gel electrophoresis analysis of positive plants using the marker gene Hyg.

Figure S2 Verification of PalbHLH1 and PalMYB90 gene expression in positive plants. **(A)** Expression level of the PalMYB90 gene. **(B)** Expression level of the PalbHLH1 gene, E=1.07. **(C)** Heights of poplar plants. **(D)** Length-to-width ratios of leaves. **(E)** Weights of poplar plants. Error bars indicate the standard deviations of five independent experiments.

Figure S3 Gene expression profiles before and after infection by two pathogens. **(A)** Expression profiles of PalbHLH1 before and after infection by two pathogens. **(B)** Expression profiles of PalMYB90 before and after infection by two pathogens. **(C)** and **(D)** Transcriptome analysis of WT and transgenic poplar. **(E)** Expression profiles of differentially expressed genes (DEGs) of WT and transgenic poplar. **(F)** DEG analysis of plants infected by pathogen compared with WT and transgenic poplar.

Figure S4 Overexpression of PalbHLH1 and PalMYB90 causes global transcriptional reprogramming in transgenic poplar. **(A)** Differential expression between MYB90/bHLH1-OE and WT plants after D. gregaria infection. **(B)** and **(C)** Transcriptome analysis of DEGs after D. gregaria infection. **(D)** Differential expression between MYB90/bHLH1-OE and WT plants after B. cinerea infection. **(E)** and **(F)** Transcriptome analysis of DEGs after B. cinerea infection.

Figure S5 Graphs displaying five standard curves. **(A)** Standard curve for determination of anthocyanin content. **(B)** Standard curve for determination of quercetin content. **(C)** Standard curve for determination of kaempferol content. **(D)** Standard curve for determination of total phenol content. **(E)** Standard curve for determination of tanin content.

Datasheets 1 Related gene accession numbers and primers for certain PCR and qPCR.

Datasheets 2 The repeated results using Deseq2 and edegR for down-regulated DEGs screening. In the data, WT group gene expression was down-regulated compared to OE group.

Datasheets 3 Read mapping results of WT, MYB90/bHLH1-OE line, WT infested by B. cinerea (WT-B. cinerea), WT infested by D. gregaria (WT-D. gregaria), MYB90/bHLH1-OE line infested by B. cinerea (OE-B. cinerea) and MYB90/bHLH1-OE line infested by D. gregaria (OE-D. gregaria).
