# Supplementary figures and images for: Coexpression of PalbHLH1 and PalMYB90 Genes From Populus alba Enhances Pathogen Resistance in Poplar by Increasing the Flavonoid Content
Source: Front Plant Sci. 2020 Feb 26;10:1772. doi: 10.3389/fpls.2019.01772 (PMC7054340; doi:10.3389/fpls.2019.01772)

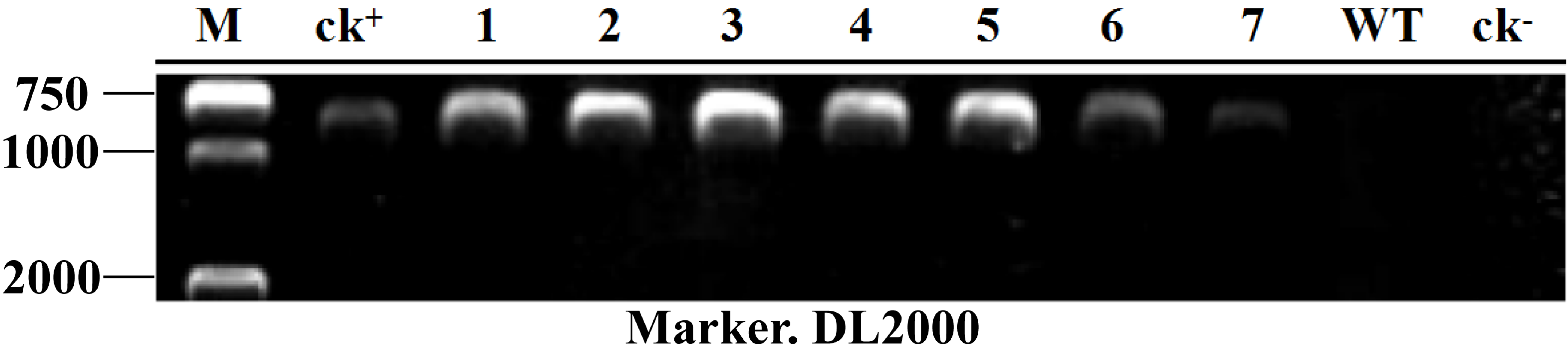

Supplement: Figure S1 — Validation of agarose gel electrophoresis analysis of positive plants using the marker gene Hyg. [file Image_1.tif]

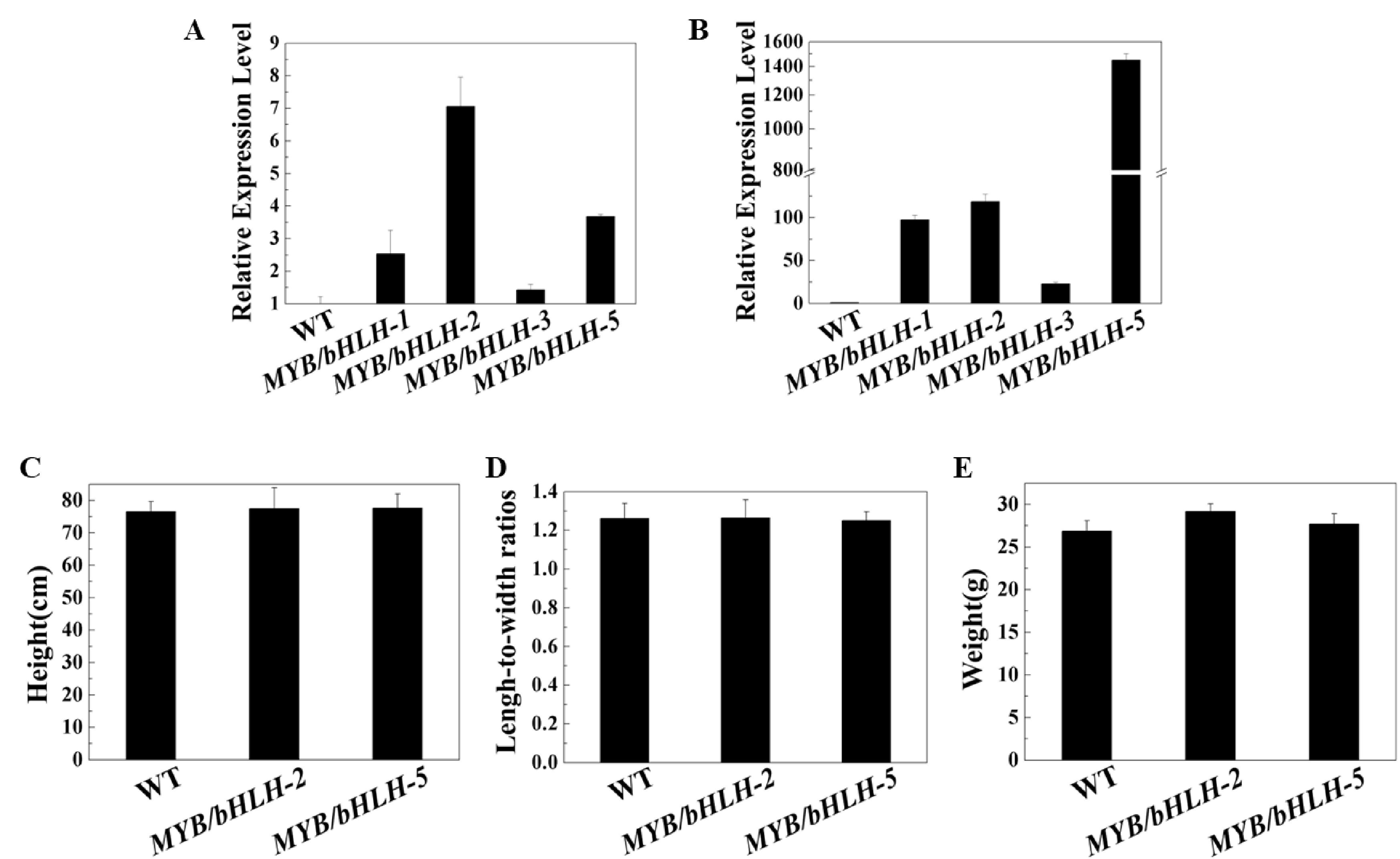

Supplement: Figure S2 — Verification of PalbHLH1 and PalMYB90 gene expression in positive plants. (A) Expression level of the PalMYB90 gene. (B) Expression level of the PalbHLH1 gene, E=1.07. (C) Heights of poplar plants. (D) Length-to-width ratios of leaves. (E) Weights of poplar plants. Error bars indicate the standard deviations of five independent experiments. [file Image_2.tif]

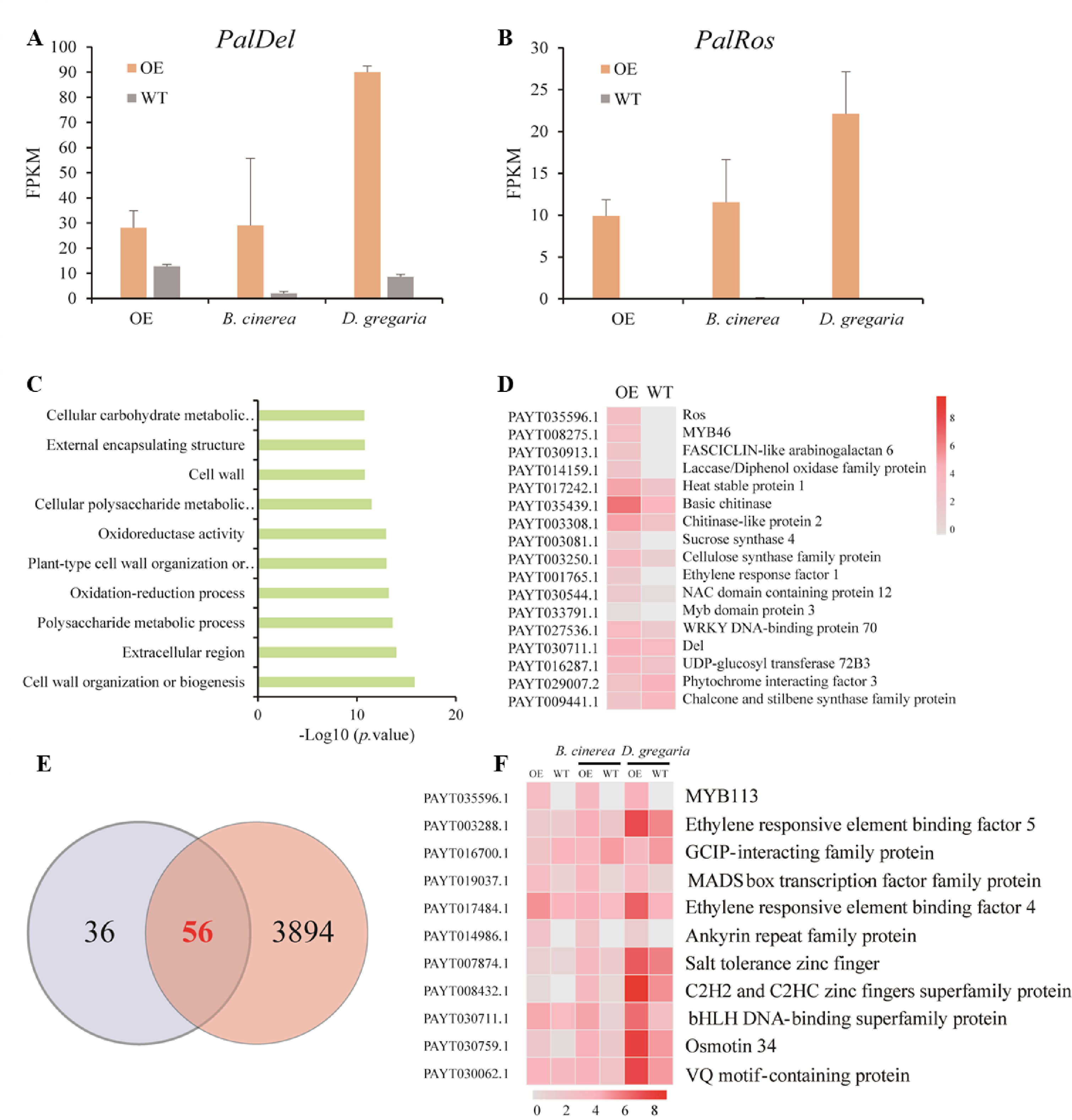

Supplement: Figure S3 — Gene expression profiles before and after infection by two pathogens. (A) Expression profiles of PalbHLH1 before and after infection by two pathogens. (B) Expression profiles of PalMYB90 before and after infection by two pathogens. (C) and (D) Transcriptome analysis of WT and transgenic poplar. (E) Expression profiles of differentially expressed genes (DEGs) of WT and transgenic poplar. (F) DEG analysis of plants infected by pathogen compared with WT and transgenic poplar. [file Image_3.tif]

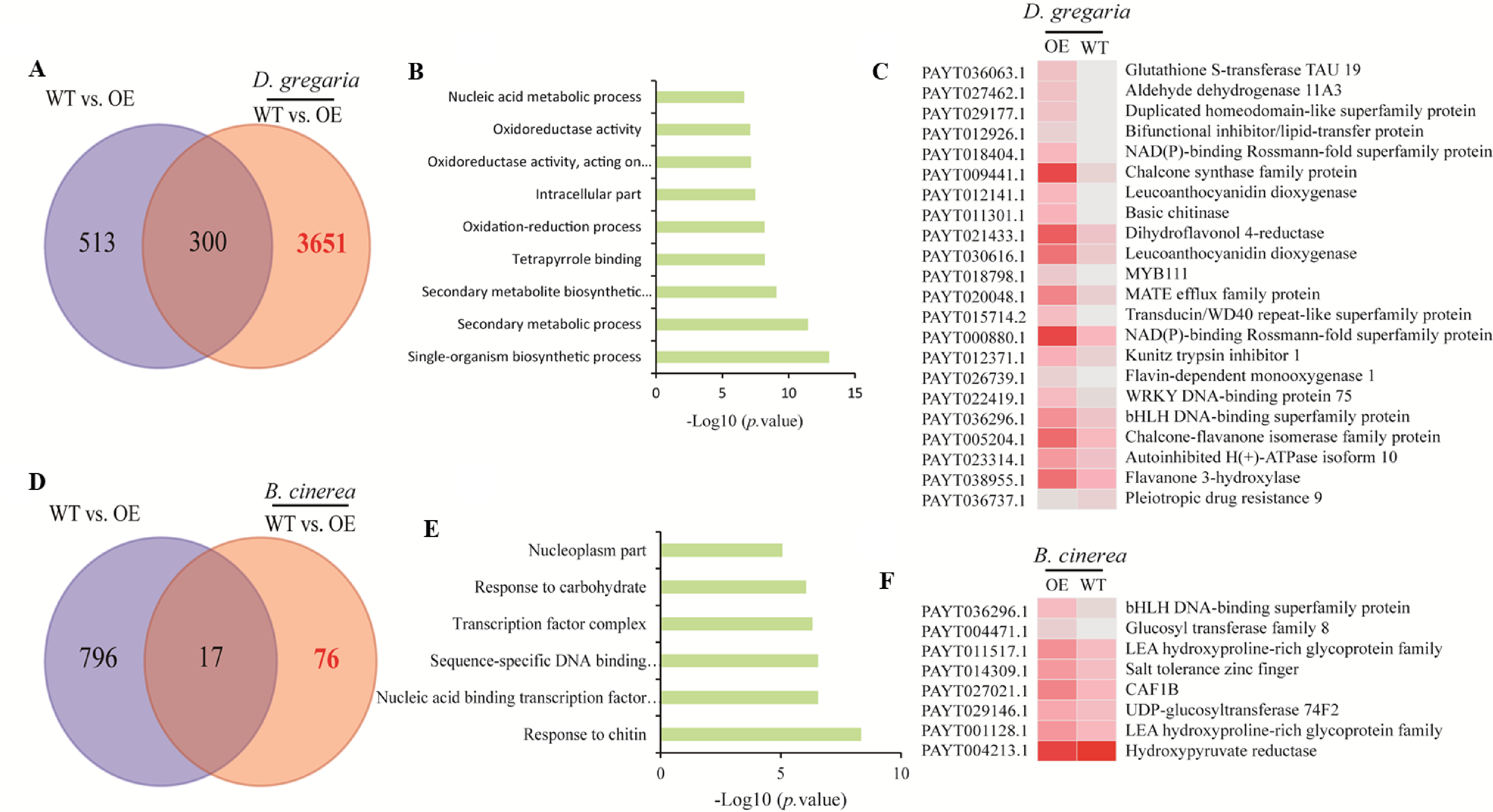

Supplement: Figure S4 — Overexpression of PalbHLH1 and PalMYB90 causes global transcriptional reprogramming in transgenic poplar. (A) Differential expression between MYB90/bHLH1-OE and WT plants after D. gregaria infection. (B) and (C) Transcriptome analysis of DEGs after D. gregaria infection. (D) Differential expression between MYB90/bHLH1-OE and WT plants after B. cinerea infection. (E) and (F) Transcriptome analysis of DEGs after B. cinerea infection. [file Image_4.tif]

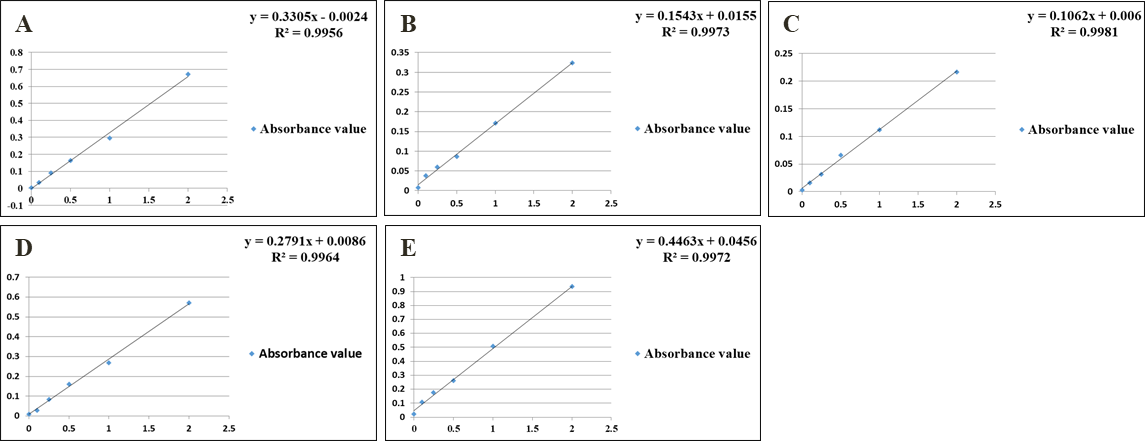

Supplement: Figure S5 — Graphs displaying five standard curves. (A) Standard curve for determination of anthocyanin content. (B) Standard curve for determination of quercetin content. (C) Standard curve for determination of kaempferol content. (D) Standard curve for determination of total phenol content. (E) Standard curve for determination of tanin content. [file Image_5.tif]
