# Supplementary material for: Coexpression of PalbHLH1 and PalMYB90 Genes From Populus alba Enhances Pathogen Resistance in Poplar by Increasing the Flavonoid Content
Source: Front Plant Sci. 2020 Feb 26;10:1772. doi: 10.3389/fpls.2019.01772 (PMC7054340; doi:10.3389/fpls.2019.01772)
Supplement: Datasheets 1 — Related gene accession numbers and primers for certain PCR and qPCR. [file DataSheet_1.doc]

TABLE S1 Names of the *PalMYB90*/ *PalbHLH1* genes in *P. alba* var. *pyramidalis* and the related structural information.

| Name | Accession Number | Size/bp | Length of CDS |
| --- | --- | --- | --- |
| *PalbHLH1* | PAYT030711.1 | 4314 | 1872 |
| *PalMYB90* | PAYT035597.1 | 3145 | 819 |

TABLE S2 Primers used for gene cloning in this study.

| Primer Name | Sequence |
| --- | --- |
| *PalbHLH1-*F | CCATCGTCAAAGTTTCTGGTGT |
| *PalbHLH1-*R | TTCTAGTGCAGAGTCCACGC |
| *PalMYB90*-F | GTCCATCCTTTGAACCATACAACC |
| *PalMYB90*-R | CAGTACGCTACAAGAGAAGCCATC |
| Hyg-1F | ATCGGACGATTGCGTCGCATC |
| Hyg-1R | GTGTCACGTTGCAAGACCTG |

TABLE S3 Primers of *PalbHLH1* and *PalMYB90* used in Quantitative real-time PCR in this study.

| Gene | Primer name | Sequence |
| --- | --- | --- |
| *PalbHLH1* | *Del* -test1F | AGTTGGTGAAGCCAGTCCAC |
|  | *Del* -test1R | AGCTAGCAGAGAGCGACTGA |
| *PalMYB90* | *Ros* -test1F | CACCTTCCGATGCGGATGAA |
|  | *Ros* -test1R | TGCTGGGTTGTCCTCTACGA |
| *CYC063* | actin-test1F | CCACGAACCGCAGAGAACTT |
|  | actin-test1R | CTCCATAGATTGATTCTCCTCCG |

TABLE S4 Primers of key genes for transcriptome analysis used in Quantitative real-time PCR in this study.

| Gene | Primer name | Sequence (5’-3’) |
| --- | --- | --- |
| *F3H* | Forward primer | GGCAACAACGGGCTCAAATC |
|  | Reverse primer | GCAATCGCAACGACCTTTGA |
| *DFR* | Forward primer | CCAACTCTTGTTGTTGGCCC |
|  | Reverse primer | AGCATCATCCGAGTTGCAGA |
| *ANS1* | Forward primer | AGGCAAGTGGATAACGGCAA |
|  | Reverse primer | CTCTGCCAATGGCTTGAGGA |
| *FLA* | Forward primer | GCCCGGTTAACTTCACCTCA |
|  | Reverse primer | TCTTGTTGGCTGAGGCCATT |
| *WRKY70* | Forward primer | TCCTTGTTATGGCCGCAAGT |
|  | Reverse primer | TCGTCGGTTAGAGTGGAGGT |
| *NAC12* | Forward primer | CTCGGCTAGAAAGCCCAACA |
|  | Reverse primer | AAGTTGCCCGGATTTGGAGT |
| *ERF1* | Forward primer | CGCCCAAGGAATCCTCAGAA |
|  | Reverse primer | CTAGCCAAACCCTGATGCCA |
| *ERF4* | Forward primer | AGCTTAATCCCAACCCCACG |
|  | Reverse primer | TAACTCCATTGGGGAACGGC |
| *ERF5* | Forward primer | GTTCACGTGTGTGGCTAGGA |
|  | Reverse primer | TTAACCACGCGCATAACCCT |
| *MYB113* | Forward primer | AAGGTGCATGGACCGAAGAG |
|  | Reverse primer | GCCAAGCAACTTGTGTAGCC |
| *LEA* | Forward primer | CCGAGTCGCCGGATTATTCA |
|  | Reverse primer | GATCCCGCGGCCATTTTAAC |
| *Prx1* | Forward primer | TGATGCGTCGGTTTTGTTGG |
|  | Reverse primer | ACTCGCAGTCTTTGAGTCCC |

TABLE S5 Accession numbers of plant bHLH proteins.

| Name | Accession Number | |
| --- | --- | --- |
| AtbHLH1 | NP_176552 |  |
| AmDel | AAA32663 |  |
| AtbHLH2 | NP_001332705 |  |
| AtTT8 | CAC14865 |  |
| AtMYC1 | AAL55719 |  |
| AtGL3 | NP_680372 |  |
| AtEGL3 | NP_974080 |  |
| PeubHLH2 | XP_011022679 |  |
| PeubHLH1 | XP_011029287 |  |
| PalbHLH1 | MK419325 |  |
| PalbHLH2 | MK419326 |  |
| PtGLABRA 3 | XP_024445100 |  |
| PtEGL1 | XP_024453227 |  |
| PhAN1 | AAG25927 |  |
| Mdbhlh33 | ABB84474 |  |
| ZmLc | NP_001105339 |  |
| VvMYCA1 | ABM92332 |  |
| IpIVS | BAF46859 |  |
| VvMYC1 | ACC68685 |  |
| PhJAF13 | AAC39455 |  |
| PfMYC-RP | BAA75513 |  |
| ZmB | CAA40544 |  |

Note: At: *Arabidopsis thaliana*; Pt: *Populus trichocarpa*; Peu: *Populus euphratica*; Pal: *Populus alba* var. *pyramidalis*; Am: *Antirrhinum majus*

Ph: *Petunia* x *hybrid*; Md: *Malus domestica*; Zm: *Zea mays*; Vv: *Vitis vinifera*; Ip: *Ipomoea purpurea*; Pf: *Perilla frutescens*

TABLE S6 Accession numbers of coding sequences of plant MYB proteins related to phenylpropanoid synthesis.

| Name | Accession Number |
| --- | --- |
| VvMYBA2 | DQ886420.1 |
| PtremMYB117 | KP723394.1 |
| PhAN2 | AF146702.1 |
| AtMYB114 | AY008379.1 |
| AtMYB113 | AY519566.1 |
| AtPAP2 | AF062915.2 |
| AtPAP1 | AY519563 |
| MtMYB5 | XM_003601561.2 |
| VvMYB5a | XM_002281607 |
| AtMYB5 | AY519587 |
| AtMYB7 | AY519573 |
| AtMYB4 | AF062860 |
| AtMYB6 | AY519604 |
| AtMYB3 | AY072543 |
| PtMYB182 | XM_002305836 |
| PtMYB165 | XM_002315854 |
| PtMYB194 | XM_002311459.2 |
| AtMYB111 | AF371977 |
| AtMYB11 | NM_116126 |
| AtMYB12 | AF062864 |
| PtMYB115 | XM_002302608.2 |
| PtMYB201 | XM_002320840.2 |
| PtMYB153 | XM_002303641.2 |
| PtMYB123 | XM_002304534.1 |
| DkMYB4 | AB503701.1 |
| PpPA1 | XM_008234893.1 |
| VvMYBPA1 | AM259485.1 |
| AtTT2 | AF371981.2 |
| FaMYB11 | JQ989282.1 |
| MdMYB11 | DQ074463.1 |
| VvPA2 | EU919682.1 |
| DkMYB2 | AB503699.1 |
| PtremMYB134 | FJ573151.1 |
| PtMYB087 | XM_002324158.2 |
| PtMYB086 | XM_006371832 |
| MdMYB9 | DQ267900.1 |
| FaMYB9 | JQ989281.1 |
| MtPAR | HQ337434.1 |
| LjTT2a | AB300033.2 |
| LjTT2c | AB300035.1 |
| LjTT2b | AB300034.2 |
| TaMYB14 | JN049641.1 |
| MtMYB14 | XM_013602969 |
| AtMYB23 | AY519631 |
| AtGL1 | AF495524 |
| PeuMYB90  PalMYB90  PtMYB90 | XM_011023092  MK419324  XP_024444020 |
| AmRos | ABB83826 |

Note: Vv: *Vitis vinifera*; Ptrem: *Populus tremula* x *Populus tremuloides*; Ph: *Petunia x hybrida*; At: *Arabidopsis thaliana*; Mt: *Medicago truncatula*; Pt: *Populus trichocarpa*; Dk: *Diospyros kaki*; Pp: *Prunus mume*; Fa: *Fragaria* x *ananassa*; Md: *Malu*s x *domestica*; Lj: *Lotus japonicus*; Ta: *Trifolium arvense*; Peu: *Populus euphratica*; Pal: *Populus alba* var. *pyramidalis*; Am: *Antirrhinum majus*

TABLE S7 Primers of structural genes of anthocyanin pathway for *P. euphratica* and *P. alba var. pyramidalis* by infecting the leaves of saplings of both with *D. gregaria* used in Quantitative real-time PCR in this study.

| Gene | Primer name | Sequence (5’-3’) |
| --- | --- | --- |
| *PeuF3H* | Forward primer | GGCAACAACGGGCTCAAATC |
|  | Reverse primer | GCAATCGCGACGACCTTTGA |
| *PeuDFR* | Forward primer | TTGGCCCATTCATCATGCCT |
|  | Reverse primer | TGCGAGGAACAGATGTAGCG |
| *PalF3H* | Forward primer | GGCAACAACGGGCTCAAATC |
|  | Reverse primer | GCAATCGCAACGACCTTTGA |
| *PalDFR* | Forward primer | CCAACTCTTGTTGTTGGCCC |
|  | Reverse primer | AGCATCATCCGAGTTGCAGA |
