# Supplementary material for: Coexpression of PalbHLH1 and PalMYB90 Genes From Populus alba Enhances Pathogen Resistance in Poplar by Increasing the Flavonoid Content
Source: Front Plant Sci. 2020 Feb 26;10:1772. doi: 10.3389/fpls.2019.01772 (PMC7054340; doi:10.3389/fpls.2019.01772)
Supplement: Supplementary file 9 [file Presentation_1.pdf]

FIGURE S1 Validation of agarose gel electrophoresis analysis of positive plants using the marker gene *Hyg*.

FIGURE S2 Verification of *PalbHLH1* and *PalMYB90* gene expression in positive plants. (A) Expression level of the *PalMYB90* gene. (B) Expression level of the *PalbHLH1* gene, E=1.07. (C) Heights of poplar plants. (D) Length-to-width ratios of leaves. (E) Weights of poplar plants. Error bars indicate the standard deviations of five independent experiments.

FIGURE S3 Gene expression profiles before and after infection by two pathogens. (A) Expression profiles of *PalbHLH1* before and after infection by two pathogens. (B) Expression profiles of *PalMYB90* before and after infection by two pathogens. (C) and (D) Transcriptome analysis of WT and transgenic poplar. (E) Expression profiles of differentially expressed genes (DEGs) of WT and transgenic poplar. (F) DEG analysis of plants infected by pathogen compared with WT and transgenic poplar.

FIGURE S4 Overexpression of *PalbHLH1* and *PalMYB90* causes global transcriptional reprogramming in transgenic poplar. (A) Differential expression between *MYB90*/*bHLH1*-OE and WT plants after *D. gregaria* infection. (B) and (C) Transcriptome analysis of DEGs after *D. gregaria* infection. (D) Differential expression between *MYB90*/*bHLH1*-OE and WT plants after *B. cinerea* infection. (E) and (F) Transcriptome analysis of DEGs after *B. cinerea* infection.

FIGURE S5 Graphs displaying five standard curves. (A) Standard curve for determination of anthocyanin content. (B) Standard curve for determination of quercetin content. (C) Standard curve for determination of kaempferol content. (D) Standard curve for determination of total phenol content. (E) Standard curve for determination of tanin content.

Datasheets 1 Related gene accession numbers and primers for certain PCR and qPCR.

Datasheets 2 The repeated results using Deseq2 and edegR for down-regulated DEGs screening. In the data, WT group gene expression was down-regulated compared to OE group.

Datasheets 3 Read mapping results of WT, *MYB90*/*bHLH1*-OE line, WT infested by *B. cinerea* (WT-*B. cinerea*), WT infested by *D. gregaria* (WT-*D. gregaria*), *MYB90*/*bHLH1*-OE line infested by *B. cinerea* (OE-*B. cinerea*) and *MYB90*/*bHLH1*-OE line infested by *D. gregaria* (OE-*D. gregaria*).
